# Stacking-dependent and electric field-driven electronic properties and band alignment transitions in γ-GeSe/Ga_2_SSe heterostructures: a first-principles study

**DOI:** 10.1039/d4na00830h

**Published:** 2024-12-12

**Authors:** Nguyen V. Vinh, D. V. Lu, K. D. Pham

**Affiliations:** a Faculty of Information Technology, Ho Chi Minh City University of Economics and Finance Ho Chi Minh City Vietnam vinhnv@uef.edu.vn; b Faculty of Physics, The University of Danang – University of Science and Education Da Nang 550000 Vietnam dvlu@ued.udn.vn; c Institute of Research and Development, Duy Tan University Da Nang 550000 Vietnam phamdinhkhang@duytan.edu.vn; d School of Engineering & Technology, Duy Tan University Da Nang 550000 Vietnam

## Abstract

In this work, we present a comprehensive investigation into the electronic properties and contact behavior of γ-GeSe/Ga_2_SSe heterostructures using first-principles calculations. Two stacking configurations, γ-GeSe/SGa_2_Se and γ-GeSe/SeGa_2_S, are explored, both exhibiting semiconducting behavior with type-II and type-I band alignments, respectively. Notably, our results show that the band alignment transition in these heterostructures can occur spontaneously by simply altering the stacking configuration, eliminating the need for external factors. Additionally, the electronic properties of these heterostructures are highly tunable with an applied electric field, further enabling transitions between type-I and type-II alignments. Specifically, a positive electric field induces a transition from type-II to type-I alignment in the γ-GeSe/SGa_2_Se heterostructure, while a negative field drives the reverse transition in the γ-GeSe/SeGa_2_S heterostructure. Our findings underscore the potential of γ-GeSe/Ga_2_SSe heterostructures for diverse applications, where the tunability of electronic properties is crucial for optimizing device performance.

## Abstract 2

In this work, we present a comprehensive investigation into the electronic properties and contact behavior of γ-GeSe/Ga_2_SSe heterostructures using first-principles calculations.

## Graphical abstract

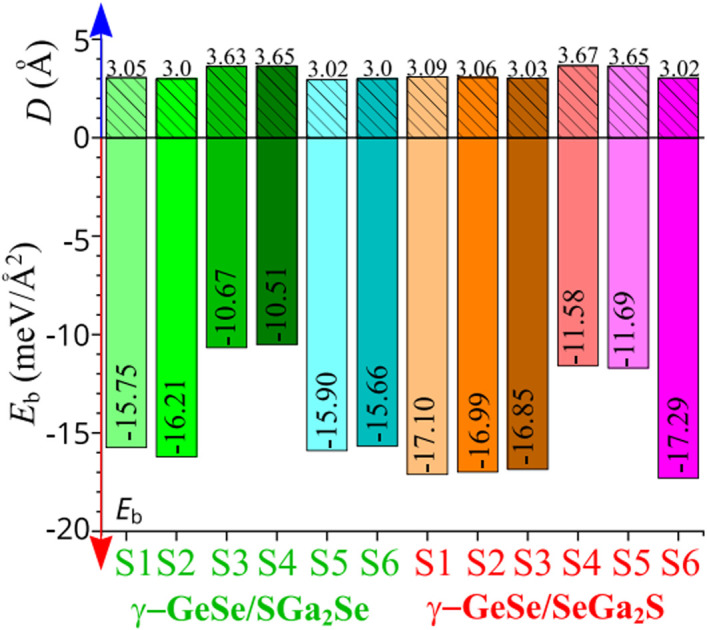

## 1 Introduction

Two-dimensional (2D) materials have garnered significant attention in recent years due to their exceptional electronic and optoelectronic properties, which arise from their atomic-scale thickness and unique crystal structures.^1–3^ Graphene,^4^ the first 2D material to be isolated and studied extensively, has served as a foundation for exploring the vast potential of this new class of materials. Following the discovery of graphene, a variety of other 2D materials have been identified, each with distinct properties that further expand the possibilities for advanced applications. Transition metal dichalcogenides (TMDs)^5,6^ exhibit tunable band gaps and strong light–matter interactions, making them suitable for optoelectronic devices and energy conversion applications. Similarly, hexagonal boron nitride (hBN) is valued for its insulating properties and chemical stability, often serving as a dielectric layer in 2D material-based devices.^7,8^ Despite the remarkable properties and potential applications of 2D materials, they also present several challenges and disadvantages that must be addressed to fully realize their potential in practical devices. For instance, the lacks in a band gap^9^ of graphene make it less suitable for applications that require a clear distinction between on and off states, such as digital transistors.^10^ In contrast, MoS_2_, a member of TMDs family, possess an intrinsic band gap,^11^ making it more suitable for semiconductor applications. However, the relatively low carrier mobility in MoS_2_ (ref. 12) compared to graphene limits its performance in high-speed electronics, where fast switching speeds and high conductivity are essential.

Recently, numerous 2D materials have continuously been discovered and successfully synthesized in experiments. Among these, a newly discovered 2D material, namely γ-GeSe,^13^ has successfully fabricated using chemical vapor deposition (CVD) method. γ-GeSe has garnered significant interest due to its predicted Mexican-hat band structure, which offers unique electronic properties.^14^ Furthermore, the electronic properties and thermoelectric performance of γ-GeSe have been found to be highly sensitive to various external conditions such as strain engineering,^15,16^ layer thickness^17^ and doping.^18,19^ The versatility in the physical properties of γ-GeSe makes it promising candidate for a wide range of applications, such as thermoelectric^13^ and energy-related^20,21^ technologies. Similar to γ-GeSe, a new family of 2D materials known as Janus 2D structures has also been recently synthesized using the CVD method.^22–24^ These Janus materials are characterized by their asymmetrical structure, where different atomic species occupy each side of the layer. The successful synthesis of Janus 2D materials marks an exciting development in the field, offering new opportunities for designing multifunctional devices with tailored properties. Alongside with the experimental achievements, various 2D Janus structures based on 2D MX_2_ and 2D MX materials have also been computationally predicted. Among these, Ga_2_SSe Janus structures have garnered interest due to its extraordinary properties, such as high carrier mobility,^25^ adjustable electronic properties under strain,^26,27^ adsorption^28^ and making vertical heterostructures.^29–31^

Currently, the integration of different 2D materials into heterostructures^32–36^ has emerged as a transformative approach for enhancing and tailoring the performance of electronic and optoelectronic devices. By stacking layers of two or more 2D materials, the 2D heterostructures can be designed with engineered band alignments and unique interfacial properties that may absent in the individual layers. The van der Waals (vdW) heterostructures, characterized by weak vdW forces rather than covalent bonds, offer precise control over electronic interactions at the interfaces, enabling the design of multifunctional devices with designed properties.^37–39^ In recent years, the focus has increasingly shifted towards integrating 2D materials with tunable electronic properties to achieve high-performance devices with enhanced functionality. The integration between γ-GeSe or Janus Ga_2_SSe material with other 2D materials have been designed previously. For example, Huan *et al.*^40^ theoretically designed the integration of γ-GeSe and 2D h-BN/graphene/MoS_2_ material. They demonstrated that the γ-GeSe/h-BN heterostructure exhibits a type-I band alignment, while the γ-GeSe/MoS_2_ heterostructure exhibits a Z-scheme type. Cao *et al.*^41^ indicated that the integration between 2D metallic NbS_2_ and γ-GeSe induces an ohmic contact, while the Bi/γ-GeSe heterostructure exhibits a Schottky contact with ultra low contact barrier. Similarly, the integration between Ga_2_SSe and other 2D materials, such as ZnO,^29^ graphene,^42^ MoSSe^43^ and silicane^44^ has been designed and extensively studied. All these heterostructures have shown promising results in enhancing electronic and optical properties, further expanding the potential of 2D materials in next-generation devices. However, the integration between γ-GeSe and Janus Ga_2_SSe materials has not yet been explored or investigated. Herein, we have designed the γ-GeSe/Ga_2_SSe heterostructure and systematically investigated its electronic properties and contact characteristics. Additionally, we explored the influence of various stacking configurations, applied electric fields, and strain engineering on the heterostructure performance. These investigations provide valuable insights into the tunability of the electronic properties and potential applications of the γ-GeSe/Ga_2_SSe heterostructure in advanced electronic and optoelectronic devices.

## 2 Computational methods

All computations were carried out using density functional theory (DFT) as implemented in the Quantum Espresso.^45^ The exchange–correlation interactions were described using the generalized gradient approximation (GGA)^46^ with the Perdew–Burke–Ernzerhof (PBE) functional.^46^ The projector augmented wave (PAW)^47^ pseudopotential was utilized for describing the electron-ion effect. To accurately describe the weak interactions between layers in the heterostructure, we employed the Grimme DFT-D3 correction method.^48^ A plane-wave basis set with an energy cutoff of 510 eV was used for all calculations to ensure reliable convergence. To prevent the interactions between periodic images of the structure, a vacuum spacing of 30 Å was introduced along the *z* direction of materials. The Brillouin zone was sampled using a 9 × 9 × 1 Monkhorst–Pack *k*-point grid. Structural relaxations were performed until the forces on each atom were less than 0.01 eV Å^−1^, and the total energy was converged to within 10^−6^ eV. The dipole corrections were also applied in all calculations. The *ab initio* molecular dynamics (AIMD) simulations were conducted using a canonical ensemble (NVT) along with the Nose–Hoover thermostat.^49^ Additionally, a 3 × 3 × 1 supercell as employed to provide adequate sampling of the system, and the simulations were performed over a duration of 5 ps with a 1 fs time step at room temperature (300 K).

## 3 Results and discussion

**Fig. 1:**
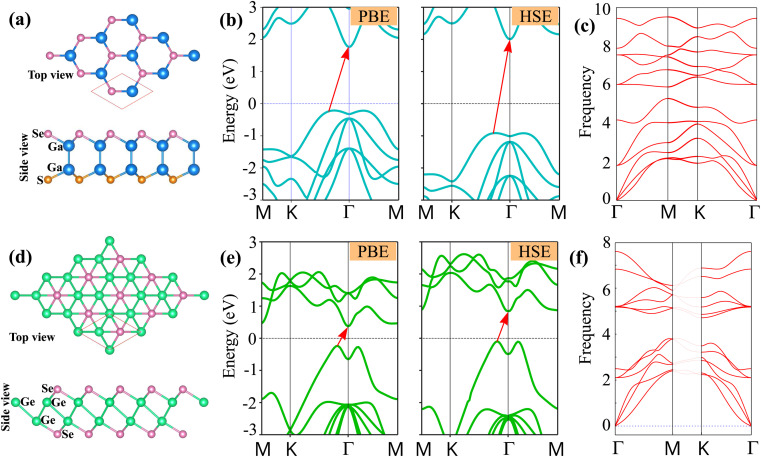
The (a and d) atomic structures, (b and e) band structures given by PBE and HSE methods and (c and f) phonon spectra of (a–c) Janus Ga_2_SSe and (d–f) γ-GeSe monolayers.

We begin by examining the atomic structures and electronic characteristics of the γ-GeSe and Janus Ga2SSe monolayers. Both materials exhibit a hexagonal lattice structure. The Janus Ga2SSe monolayer is classified under the point group *C*_3v_ (*P*3*m*1), while the γ-GeSe monolayer is categorized under the point group *C*_6v_ (6*mm*). Each unit cell of the Janus Ga_2_SSe monolayer contains a total of four atoms, comprising two gallium (Ga) atoms, one sulfur (S) atom, and one selenium (Se) atom. An unit cell of the γ-GeSe monolayer consists of two germanium (Ge) atoms and two Se atoms. Both Ga_2_SSe and γ-GeSe monolayers are classified as indirect semiconductors, with the conduction band minimum (CBM) located at the *Γ* point and the valence band maximum (VBM) found along the *K*–*Γ* path. The obtained band gap of γ-GeSe is 0.57 eV predicted by PBE method and 1.0 eV given by HSE method. While the Ga_2_SSe monolayer shows a larger band gap of 2.07 eV for PBE and 2.98 eV for HSE method. It is well established that the HSE method offers a more accurate estimation of the band gap compared to PBE. However, PBE remains a reasonable approach for investigating trends and qualitative behavior in both the γ-GeSe and Ge_2_SSe monolayers as well as their corresponding heterostructures. This is because the consistency between the HSE-calculated results for those monolayers and the trends observed using PBE lends confidence to the reliability of PBE for capturing the electronic properties in such materials. Furthermore, the stability of both monolayers is confirmed by the absence of imaginary frequencies in their phonon spectra at the *Γ* point, as illustrated in Fig. 1(c) and (f). The obtained lattice constants of γ-GeSe and Ga_2_SSe monolayers are 3.74 and 3.72 Å, respectively, as illustrated in Table 1. These values are in good agreement with the previous reports,^25,50^ confirming the reliability and accuracy of our computational methods. Based on the above findings, we decided to select the PBE method for subsequent calculations, as it provides reliable trends and qualitative insights while maintaining computational efficiency.

**Table 1:** Calculated lattice constant (*a* Å), interlayer distance (*D* Å), binding energy (*E*_b_ meV Å^−2^), band gap (*E*_g_ eV), band nature and band alignment of the γ-GeSe/Ga_2_SSe heterostructure

		*a*	*D*	*E*_b_	*E*_g_	Band nature	Band alignment
Monolayers	γ-GeSe	3.74	—	—	0.57	Indirect	—
	Ga_2_SSe	3.72	—	—	2.07	Indirect	—
γ-GeSe/SGa_2_Se	S1	3.72	3.06	−15.75	0.78	Indirect	Type-II
	S2	3.72	3.0	−16.21	0.78	Indirect	Type-II
	S3	3.72	3.63	−10.67	0.78	Indirect	Type-II
	S4	3.72	3.65	−10.51	0.78	Indirect	Type-II
	S5	3.72	3.02	−15.90	0.78	Indirect	Type-II
	S6	3.72	3.0	−15.66	0.78	Indirect	Type-II
γ-GeSe/SeGa_2_S	S1	3.72	3.09	−17.10	0.77	Indirect	Type-I
	S2	3.72	3.06	−16.99	0.77	Indirect	Type-I
	S3	3.72	3.03	−16.85	0.77	Indirect	Type-I
	S4	3.72	3.67	−11.58	0.77	Indirect	Type-I
	S5	3.72	3.65	−11.66	0.77	Indirect	Type-I
	S6	3.72	3.02	−17.29	0.77	Indirect	Type-I

**Fig. 2:**
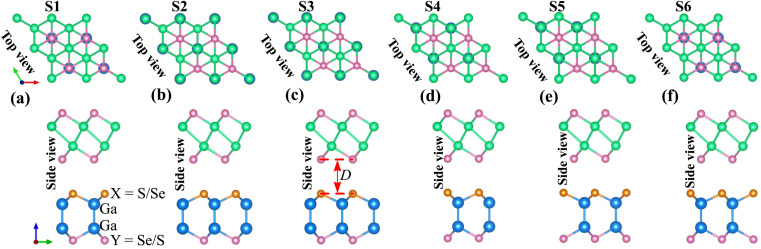
Top view and side view of the γ-GeSe/Ga_2_SSe heterostructures for six different arrangements of (a) S1, (b) S2, (c) S3, (d) S4, (e) S5 and (f) S6 stacking.

**Fig. 3:**
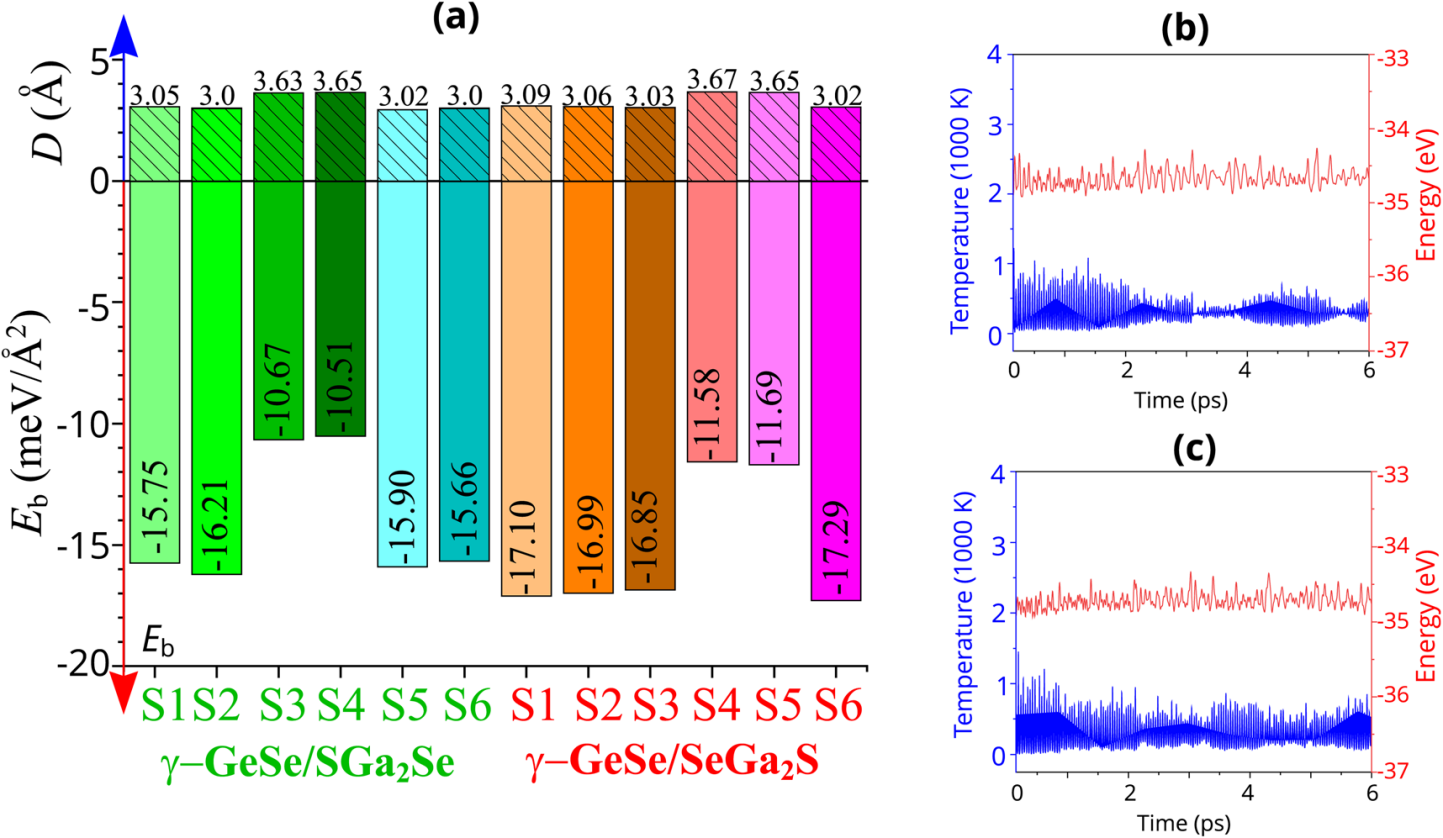
(a) Calculated binding energies and interlayer spacings in the γ-GeSe/Ga_2_SSe heterostructures for different arrangements. AIMD simulation of the total energy and temperature of the most energetically favorable arrangement (b) S2 for γ-GeSe/SGa_2_Se and (c) S6 for γ-GeSe/SeGa_2_S heterostructure.

The atomic structures of the γ-GeSe/Ga_2_SSe heterostructure are displayed in Fig. 2. Because of the asymmetrical structure in Janus Ga_2_SSe, the γ-GeSe/Ga_2_SSe heterostructure results in the formation of two distinct stacking configurations: γ-GeSe/SGa_2_Se and γ-GeSe/SeGa_2_S. In the γ-GeSe/SGa_2_Se configuration, the γ-GeSe layer is placed above on top of the sulfur (S) layer of Ga_2_SSe, while in the γ-GeSe/SeGa_2_S configuration, the γ-GeSe layer is above on top of the selenium (Se) layer of Ga_2_SSe layer. Additionally, each γ-GeSe/SGa_2_Se or γ-GeSe/SeGa_2_S configuration consists of totally six different stacking patterns, as illustrated in Fig. 2. Additionally, the lattice mismatch between the γ-GeSe and Ga_2_SSe is obtained to be less than 3%. The interlayer spacing *D* between the lowest Se layer of the γ-GeSe layer and the highest S/Se layer of the Ga_2_SSe layer can be obtained after the geometric optimization. The obtained *D* are illustrated in Fig. 3(a), showing a range from 3 to 3.67 Å. For the γ-GeSe/SGa_2_Se heterostructure, the shortest interlayer spacing occurs in the S2 stacking, while for the γ-GeSe/SeGa_2_ heterostructure, the S6 stacking exhibits the shortest interlayer spacing. Interestingly, these values of the interlayer spacings are comparable with those of the other heterostructures, such as Ga_2_SSe/GaN,^31^ g-CN/Mo(W)Te_2_,^51^ graphene/MoSi_2_N_4_ (ref. 52) MoTe_2_/MoS_2_,^53^ MX (M = Ga, In; X = S, Se, Te)/GaInS_3_ (ref. 54) BX–SiS (X = As, P)^55^ and NbS_2_/BSe.^56^ This observation shows that the γ-GeSe and Ga_2_SSe layer interact through the weak interactions.

Furthermore, to access the stability of the γ-GeSe/Ga_2_SSe heterostructure, we perform the binding energy calculations as the difference in the total energies of the heterostructure and the isolated monolayers as below:1
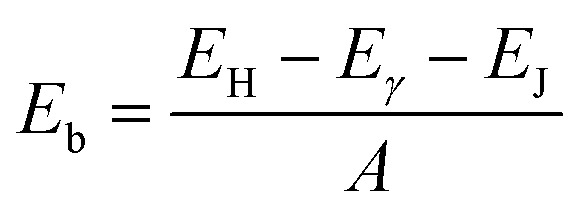


The binding energy of the γ-GeSe/Ga_2_SSe heterostructure for all stacking patterns are depicted in Fig. 3(a). Notably, the binding energy *E*_b_ ranges from −10 to −18 meV Å^−2^. The negative values of the *E*_b_ indicate that this heterostructure is stable. Interestingly, the *E*_b_ values are comparable with those in other vdW-typical systems, such as graphite,^57,58^ graphene/InSe^59^ and MX_2_ compounds.^60^ This observation indicates that the γ-GeSe/Ga_2_SSe heterostructures are mainly governed by physicoadsorption rather than strong chemical bonding. This type of interaction often leads to stable and further support the stability of the heterostructure while allowing for potential flexibility in its properties. For the γ-GeSe/SGa_2_Se heterostructure, the S2 stacking shows the lowest value of the *E*_b_, specializing that it is the most energetically favorable arrangement. Similarly, in the γ-GeSe/SeGa_2_S heterostructure, the S6 stacking exhibits the lowest binding energy, signifying that this configuration is the most energetically stable. Hence, the S2 stacking of the γ-GeSe/SGa_2_Se and S6 stacking of the γ-GeSe/SeGa_2_S heterostructures will be the focus of subsequent investigations. The *ab initio* molecular dynamics (AIMD) simulations of the total energy and temperature are performed to access the thermal stability of the most energetically favorable arrangement of the S2 stacking in the γ-GeSe/SGa_2_Se and S6 stacking in the γ-GeSe/SeGa_2_S heterostructures. These results of the AIMD simulations are depicted in Fig. 3(b) and (c). Notably, the small variations in total energies and temperatures during the relaxation process suggest that the heterostructures exhibit thermal stability.

**Fig. 4:**
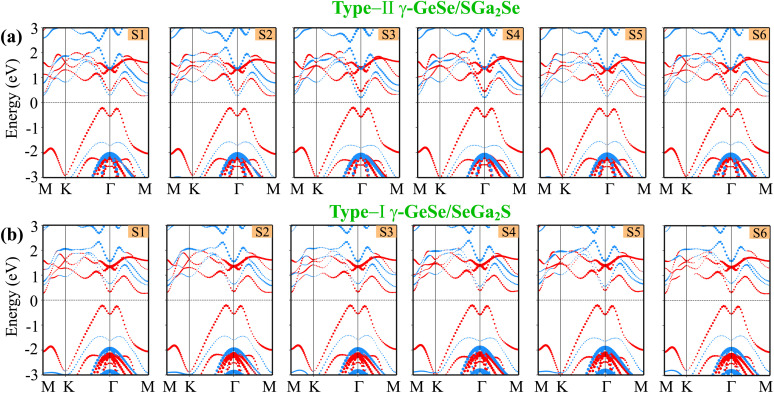
The projections of the band structures for the different stacking arrangements in the (a) γ-GeSe/SGa_2_Se and (b) γ-GeSe/SeGa_2_S heterostructures. The projections of the γ-GeSe and Ga_2_SSe layers are visualized by red and blue lines, respectively.

We further examine the electronic properties of the γ-GeSe/Ga_2_SSe heterostructures for various stacking arrangements. The projections of the band structures of the γ-GeSe/SGa_2_Se heterostructure for six stacking arrangements are illustrated in Fig. 4(a). It can be seen that the γ-GeSe/SGa_2_Se heterostructure exhibits the semiconducting features with the indirect band gap nature. The VBM is located at the *Γ* point and the CBM is located along the *K*–*Γ* path. The band gap of the γ-GeSe/SGa_2_Se heterostructure for six stacking arrangements is obtained to be 0.78 eV. This value is smaller than that of the Ga_2_SSe material, but it is larger than that of the γ-GeSe material. Notably, the indirect band gap characteristic of the heterostructure makes it promising for photodetection and solar energy conversion. Additionally, we observe that the contribution of the γ-GeSe and Ga_2_SSe layers to the band edges of their heterostructure varies across different stacking arrangements, as indicated by the weighted projections in Fig. 4(a). In all six stacking arrangements of the γ-GeSe/SGa_2_Se heterostructure, the VBM is dominated by contributions from the γ-GeSe layer, while the CBM is mainly derived from the Ga_2_SSe layer, specifying the type-II band alignment. This type of alignment is beneficial for applications such as solar cells and photodetectors, where efficient charge separation is crucial.

Similarly, the γ-GeSe/SeGa_2_S heterostructure also exhibits the semiconducting nature with an indirect band gap, as depicted in Fig. 4(b). In this case, VBM is located at the *Γ* point, while the CBM is positioned along the *Γ*–*K* path. The indirect nature of the band gap can influence the efficiency of optoelectronic devices, potentially lowering their performance in applications like light-emitting diodes (LEDs), but it may still be advantageous for other applications such as thermoelectrics^61–63^ More interestingly, the γ-GeSe/SeGa_2_S heterostructure forms a type-I band alignment, where the band edges of the γ-GeSe layer lie within the band edges of the Ga_2_SSe. It indicates that both the VBM and CBM of the γ-GeSe/SeGa_2_S heterostructure is mainly contributed by the γ-GeSe layer. One should be pointed out that this type of band alignment is advantageous for optoelectronic applications such as light-emitting diodes (LEDs) and lasers because it efficiently confines both electrons and holes within the same material layer, facilitating enhanced recombination. Hence, type-I band alignment is highly advantageous for light-emitting applications because it traps both electrons and holes in a region where efficient radiative recombination can occur, making it ideal for LEDs, lasers, and other optoelectronic devices that rely on light emission.^64,65^ The coexistence of both type-I and type-II band alignments in the γ-GeSe/Ga_2_SSe heterostructure demonstrates the versatility of these materials. By selecting different stacking configurations, it is possible to tailor the electronic and optoelectronic properties of the heterostructure for specific applications, ranging from energy-harvesting devices to light-emitting components.

Furthermore, the charge transfers between the γ-GeSe and Ga_2_SSe layers are visualized by considering the charge density difference (CDD) as:

2 Δ*ρ* = *ρ*_H_ − *ρ*_*γ*_ − *ρ*_G_

**Fig. 5:**
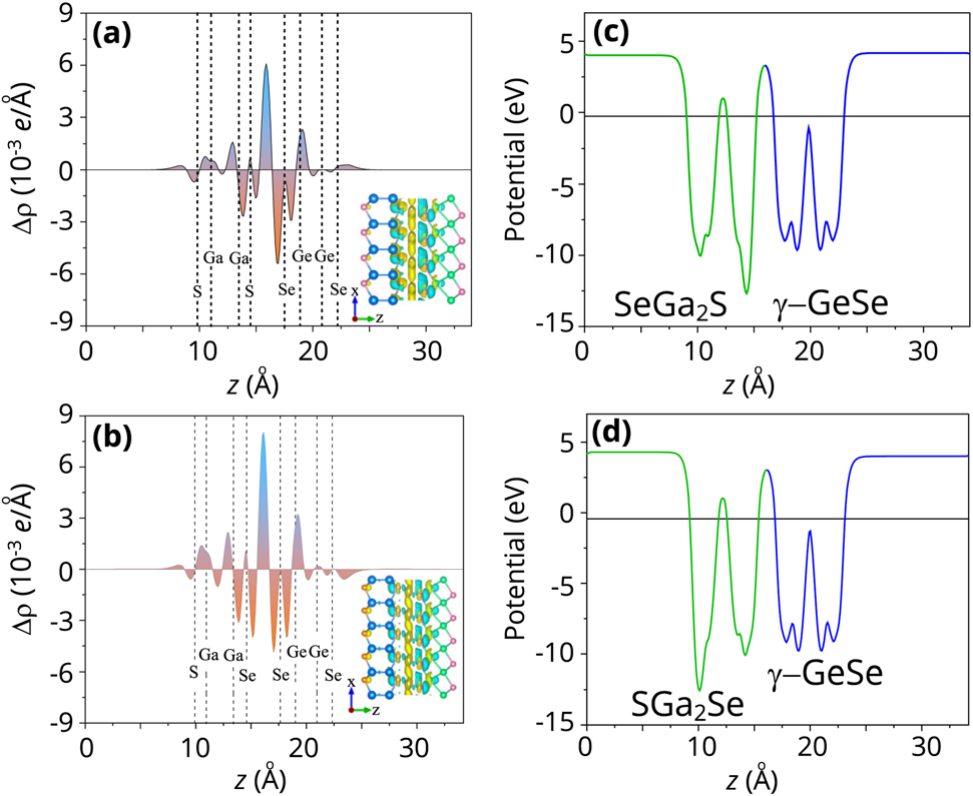
(a) Charge density difference and (c and d) electrostatic potential of the (a and c) γ-GeSe/SGa_2_Se and (b and d) γ-GeSe/SeGa_2_S heterostructures. The yellow and cyan areas indicate the positive and negative charges, respectively.

Here, *ρ*_*γ*_, *ρ*_G_ and *ρ*_H_ are the charge densities of the isolated γ-GeSe, Ga_2_SSe and the γ-GeSe/Ga_2_SSe heterostructures, respectively. The CDD of the γ-GeSe/SGa_2_Se and γ-GeSe/SeGa_2_S heterostructures are displayed in Fig. 5(a) and (b). For the γ-GeSe/SGa_2_Se heterostructure, the positive and negative charges indicate the charge accumulation and depletion, respectively. We can find that the positive charges are observed in the side of the S layer in the Ga_2_SSe layer, while the negative charges are mainly occurred in the side of the Se layer in the γ-GeSe layer. This observation means that the electrons are transferred from the Ga_2_SSe layer to the γ-GeSe layer. The Ga_2_SSe act as electron donors with the electron depletion, whereas the γ-GeSe receive the electrons with the charge accumulation. The similar observation also occurs in the γ-GeSe/SeGa_2_S heterostructure. In this configuration, positive charges accumulate on the Se side of the Ga_2_SSe layer, while negative charges are concentrated on the γ-GeSe layer. This indicates that electrons are transferred from the Ga_2_SSe layer to the γ-GeSe layer. Bader charge analysis indicates that only 0.0015 electrons are transferred at the interface of the γ-SGa_2_Se heterostructure, while 0.003 electrons are transferred in the γ-GeSe/SeGa_2_Se heterostructure. One should be noted that the charge redistribution across the interface leads to the formation of a dipole layer. The electrostatic potentials of the heterostructures are depicted in Fig. 5(c) and (d). It is evident that there is a noticeable potential difference between the two layers in both the γ-GeSe/SGa_2_Se and γ-GeSe/SeGa_2_S configurations. This potential difference is a clear indicator of the built-in electric field at the interface, which arises due to the charge redistribution between the two layers. In both these arrangements, the potential of the γ-GeSe layer is higher than that of the Ga_2_SSe layer, confirming that electron transfer occurs from the Ga_2_SSe layer to the γ-GeSe layer.

Moreover, the transport properties of the γ-GeSe/Ga_2_SSe heterostructures are also calculated to confirm their potential applications in next-generation electronic and optoelectronic devices. The carrier mobility of heterostructures can be obtained as follows:3
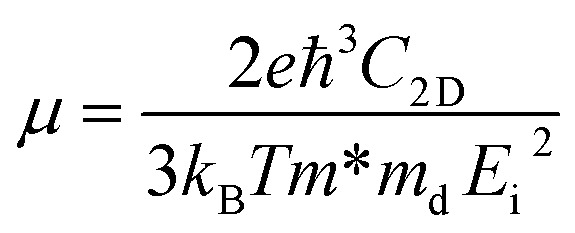
Here, *e* and m* is the charge and effective mass, respectively. *k*_B_ is the Boltzmann's constant. *m*_d_ is the equivalent mass, which is obtained as 
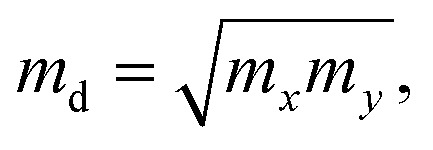
*E*_i_ is the deformation potential for electrons or holes. The carrier mobilities of the γ-GeSe and Ga_2_SSe monolayers are also calculated for the comparison. Our results demonstrate that the γ-GeSe and Ga_2_SSe monolayers exhibit high carrier mobilities of approximately 812/421 and 303/141 cm^2^ V^−1^ s^−1^ for electrons/holes, respectively, which align well with previously reported values.^14,44^ Upon forming the γ-GeSe/Ga_2_SSe heterostructure, the carrier mobility significantly increases, reaching 2105/709 cm^2^ V^−1^ s^−1^ in the γ-GeSe/SGa_2_Se configuration and 3217/767 cm^2^ V^−1^ s^−1^ in the γ-GeSe/SeGa_2_S configuration for electrons/holes. These values of the carrier mobility are comparable with those in other heterostructures, such as BC_6_N/BN,^66^ MoSSe/GaN,^67^ Sb/SnSe^68^ and GeC(ZnO)/Al_2_SO^69^ heterostructures. The enhancement in the carrier mobility suggests that the formation of the γ-GeSe/Ga_2_SSe heterostructure significantly improves the electronic transport properties, making it highly advantageous for high-performance electronic and optoelectronic applications.

**Fig. 6:**
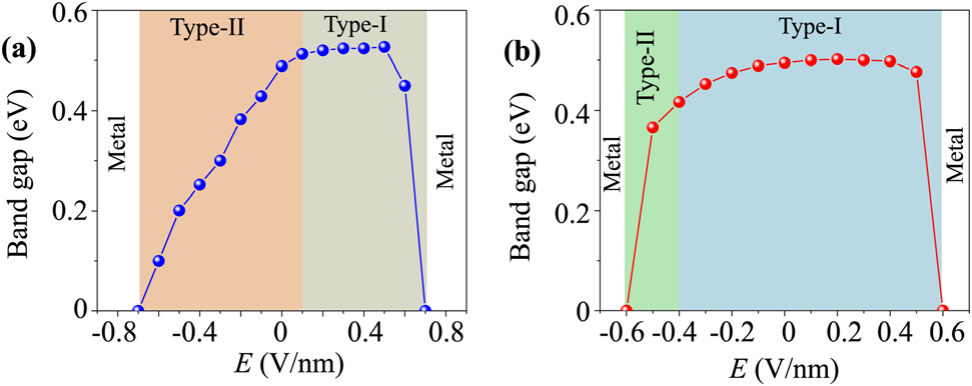
The variation of the band gap of (a) γ-GeSe/SGa_2_Se and (b) γ-GeSe/SeGa_2_S heterostructure under applied electric field.

More interestingly, the versatility in the electronic properties of the γ-GeSe/Ga_2_SSe heterostructure are crucial for the practical application. The ability to modulate band alignments of the heterostructure opens up opportunities for designing multifunctional devices. Therefore, we further investigate the versatility in the electronic properties of the γ-GeSe/Ga_2_SSe heterostructure under applied electric fields. The variations in the band gap of the γ-GeSe/Ga_2_SSe heterostructure under applied electric field are presented in Fig. 6. It is evident that the electric fields vary the band gap of the γ-GeSe/Ga_2_SSe heterostructure and include the transformation between type-I and type-II band alignment. For the γ-GeSe/SGa_2_Se heterostructure, the negative electric field results in a reduction of the band gap. When a negative electric field of −0.7 V nm^−1^ is applied, the band gap of the heterostructure reduces to zero, causing a transition from semiconductor to metal. On the contrary, applying a positive electric field initially causes the band gap to increase. However, when the positive electric field exceeds +0.5 V nm^−1^, a reversal is observed, leading to a gradual decrease in the band gap. When a positive electric field of +0.7 V nm^−1^ is applied, the band gap of the heterostructure closes, resulting in a transition to a metallic state. The transformation from type-II to type-I is observed in the γ-GeSe/SGa_2_Se heterostructure under the positive electric field of +0.1 V nm^−1^. For the γ-GeSe/SeGa_2_S heterostructure, a similar behavior is observed under an applied electric field. Both negative and positive electric fields drive the transition of the heterostructure from a semiconductor to a metallic state. Specifically, a negative electric field causes a reduction in the band gap, while a positive electric field initially enhances the band gap. However, when the positive electric field exceeds +0.4 V nm^−1^, the band gap begins to decrease again. Additionally, the application of electric fields not only alters the band gap but also induces a transformation in the band alignment. Under a negative electric field lower than −0.4 V nm^−1^, the band alignment shifts from type-I to type-II, further emphasizing the tunability of the electronic properties of the heterostructure. This flexibility in adjusting both band gap and band alignment highlights the potential of the γ-GeSe/Ga_2_SSe heterostructure for multifunctional device applications.

**Fig. 7:**
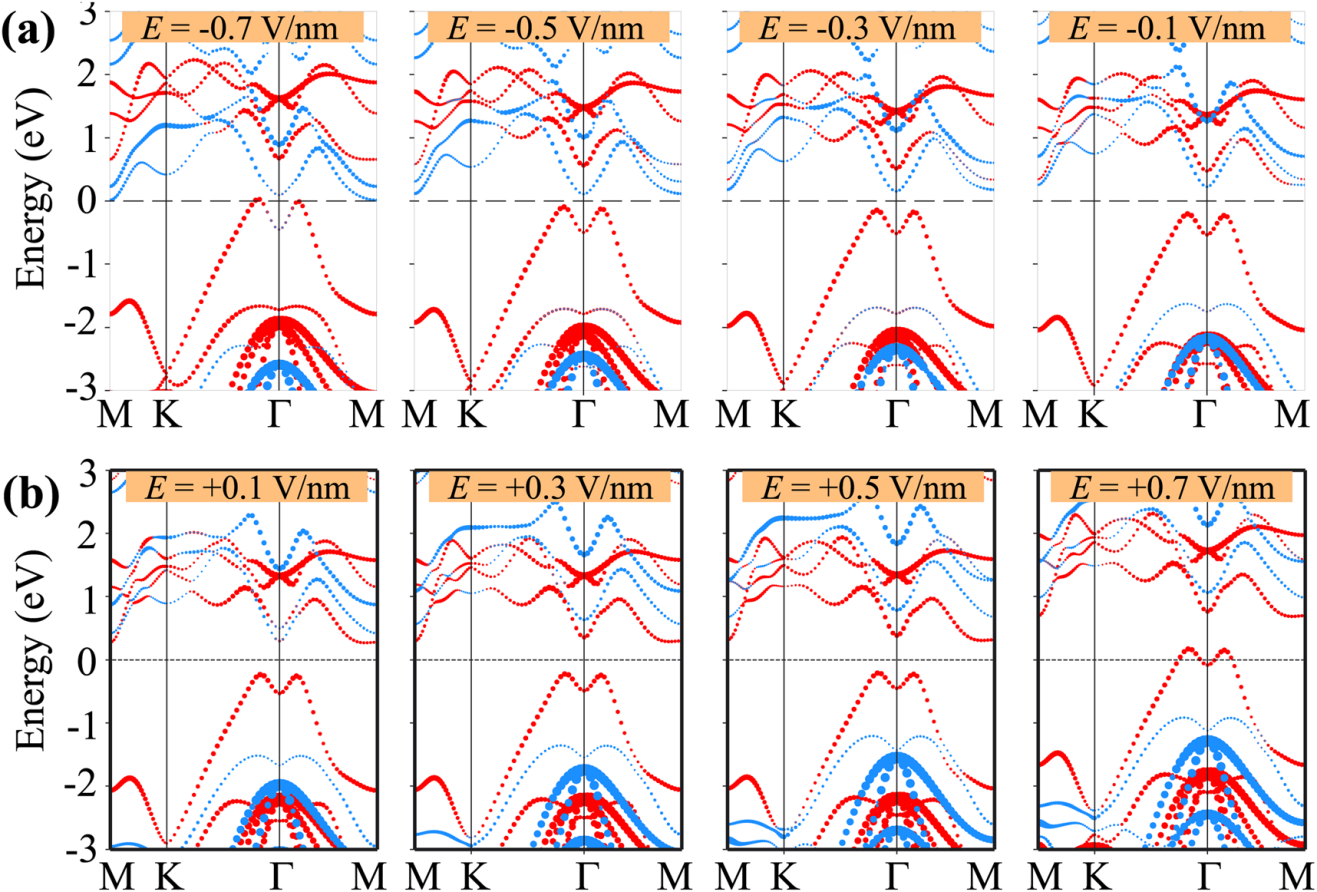
Weighted projections of the band structures of the γ-GeSe/SGa_2_Se heterostructure under applied (a) negative and (b) positive electric fields. The projections of the γ-GeSe and Ga_2_SSe layers are presented by the red and blue balls, respectively.

**Fig. 8:**
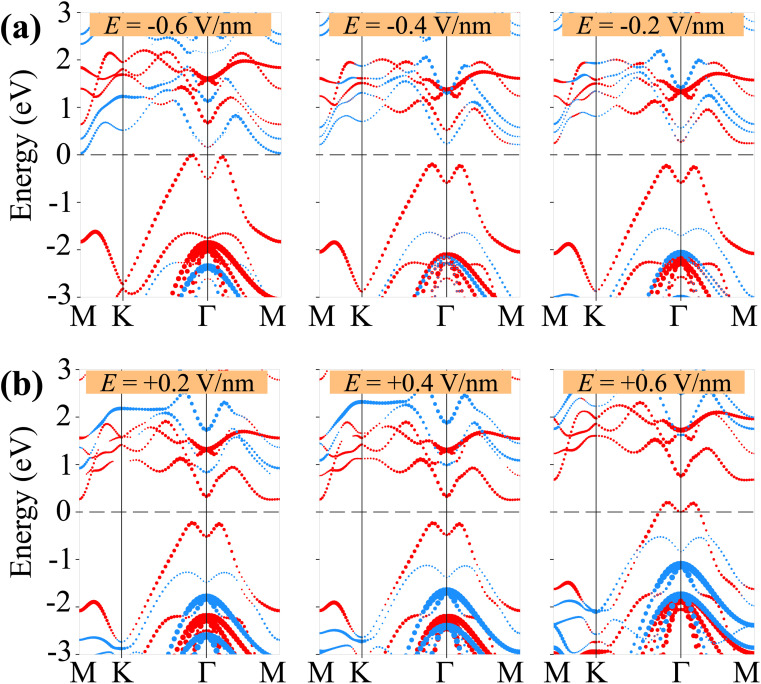
Weighted projections of the band structures of the γ-GeSe/SeGa_2_S heterostructure under applied (a) negative and (b) positive electric fields. The projections of the γ-GeSe and Ga_2_SSe layers are presented by the red and blue balls, respectively.

We further analyze the weighted projections of the band structures for both the γ-GeSe/SGa_2_Se and γ-GeSe/SeGa_2_S heterostructures under varying strengths of applied electric fields, as shown in Fig. 7 and 8. This detailed investigation reveals the underlying mechanisms responsible for the tunability of the electronic properties. It is evident that the negative electric field induces the shifts in the band edges of the γ-GeSe and Ga_2_SSe layers in two opposite directions. The band edges of the γ-GeSe shift towards the region with the higher binding energy, while the band edges of the Ga_2_SSe layer shifts in the opposite direction, *i.e.* toward the region with the lower binding energy. The shifts are depicted in Fig. 7(a). This observation also supports that the band gap of the γ-GeSe/SGa_2_Se heterostructure is reduced upon the application of the negative electric field. The band edges of the heterostructure cross the Fermi level under the negative electric field of −0.7 V nm^−1^, driving the transition from the semiconductor to metal. Similarly, when a positive electric field is applied, the band edges of the two constituent layers shift in opposite directions. Under a positive electric field, the band edges of the γ-GeSe layer shift downward toward lower binding energies, while the band edges of the Ga_2_SSe layer shift upward toward higher binding energies. This opposing behavior between the layers contributes to further modulation of the electronic properties, including changes in the band gap and type of band alignment. Under the positive electric field exceeded +0.1 V nm^−1^, the band edges of the γ-GeSe/SGa_2_Se heterostructure are mainly derived from the γ-GeSe layer, signifying a transformation from type-II to type-I. When a positive electric field of +0.7 V nm^−1^ is applied, the VBM of the heterostructure crosses the Fermi level, signaling a transition from a semiconductor to a semimetal. The shift in the band alignment of the γ-GeSe/SGa_2_Se heterostructure, driven by the applied electric field, suggests its potential as a promising candidate for multifunctional devices. The similar shifts in the band edges of the constituent γ-GeSe and Ga_2_SSe layer under applied electric fields are also observed in the γ-GeSe/SeGa_2_S heterostructure, as illustrated in Fig. 8. Notably, a transformation from type-I to type-II band alignment occurs under a negative electric field of −0.4 V nm^−1^. In this scenario, the VBM is dominated by contributions from the γ-GeSe layer, while the CBM originates from the Ga_2_SSe layer, signifying a clear conversion to type-II band alignment. Furthermore, the band edges of the heterostructure cross the Fermi level at the electric field of *E* = ±0.6 V nm^−1^, confirming the transition from semiconductor to metallic state. Therefore, the tunability in electronic properties, particularly the ability to transition between type-I and type-II band alignments in the γ-GeSe/SGa_2_Se and γ-GeSe/SeGa_2_S heterostructures, opens up new possibilities for diverse applications, including transistors, photodetectors, and optoelectronic devices.

## 4 Conclusions

In conclusion, we have systematically explored the electronic properties and contact behavior of the γ-GeSe/Ga_2_SSe heterostructures using first-principles calculations. Our findings reveal that the heterostructures exhibit tunable electronic characteristics, with both the γ-GeSe/SGa_2_Se and γ-GeSe/SeGa_2_S configurations demonstrating semiconducting behavior. Notably, the γ-GeSe/SGa_2_Se heterostructure forms a type-II band alignment, while the γ-GeSe/SeGa_2_S heterostructure exhibits a type-I band alignment. The γ-GeSe/Ga_2_SSe heterostructures demonstrate significantly enhanced carrier mobility compared to their constituent monolayers. The application of an external electric field significantly modulates the band alignment and electronic properties of the heterostructures. A positive electric field induces a transition from type-II to type-I band alignment in the γ-GeSe/SGa_2_Se heterostructure, while a negative electric field causes the opposite transformation in the γ-GeSe/SeGa_2_S heterostructure. The versatility in tuning electronic properties underscores the potential of the γ-GeSe/Ga_2_SSe heterostructures for next-generation multifunctional devices. These findings provide a promising foundation for future research and development in 2D material-based heterostructures, enabling advanced device engineering with enhanced performance and functionality.

## Data availability

The data that support the findings of this study are available from the corresponding author upon reasonable request.

## Conflicts of interest

There are no conflicts to declare.
